# Preclinical models of mitochondrial dysfunction: mtDNA and nuclear-encoded regulators in diverse pathologies

**DOI:** 10.3389/fragi.2025.1585508

**Published:** 2025-06-23

**Authors:** Dalia M. Miller, Stephen L. Archer, Kimberly J. Dunham-Snary

**Affiliations:** ^1^ Department of Medicine, Queen’s University, Kingston, ON, Canada; ^2^ Queen’s CardioPulmonary Unit, Queen’s University, Kingston, ON, Canada; ^3^ Department of Medicine, Translational Institute of Medicine, Queen’s University, Kingston, ON, Canada; ^4^ Department of Biomedical and Molecular Science, Queen’s University, Kingston, ON, Canada

**Keywords:** mitochondrial-driven diseases, preclinical models, cybrid, conplastic mouse, mitochondrial-nuclear eXchange (MNX) mice, mitochondrial replacement therapy (MRT), CRISPR/Cas9, organoid

## Abstract

Mitochondrial-driven diseases encompass a diverse group of single-gene and complex disorders, all linked to mitochondrial dysfunction, with significant impacts on human health. While there are rare mitochondrial diseases in which the primary defect resides in mutations in mitochondrial DNA, it is increasingly clear that acquired mitochondrial dysfunction, both genetically- and epigenetically-mediated, complicates common complex diseases, including diabetes, cardiovascular disease and ischemia reperfusion injury, cancer, pulmonary hypertension, and neurodegenerative diseases. It is also recognized that mitochondrial abnormalities not only act by altering metabolism but, through effects on mitochondrial dynamics, can regulate numerous cellular processes including intracellular calcium handling, cell proliferation, apoptosis and quality control. This review examines the crucial role of preclinical models in advancing our understanding of mitochondrial genetic contributions to these conditions. It follows the evolution of models of mitochondrial-driven diseases, from earlier *in vitro* and *in vivo* systems to the use of more innovative approaches, such as CRISPR-based gene editing and mitochondrial replacement therapies. By assessing both the strengths and limitations of these models, we highlight their contributions to uncovering disease mechanisms, identifying therapeutic targets, and facilitating novel discoveries. Challenges in translating preclinical findings into clinical applications are also addressed, along with strategies to enhance the accuracy and relevance of these models. This review outlines the current state of the field, the future trajectory of mitochondrial disease modeling, and its potential impact on patient care.

## 1 Introduction

### 1.1 Mitochondrial-driven diseases

**FIGURE 1:**
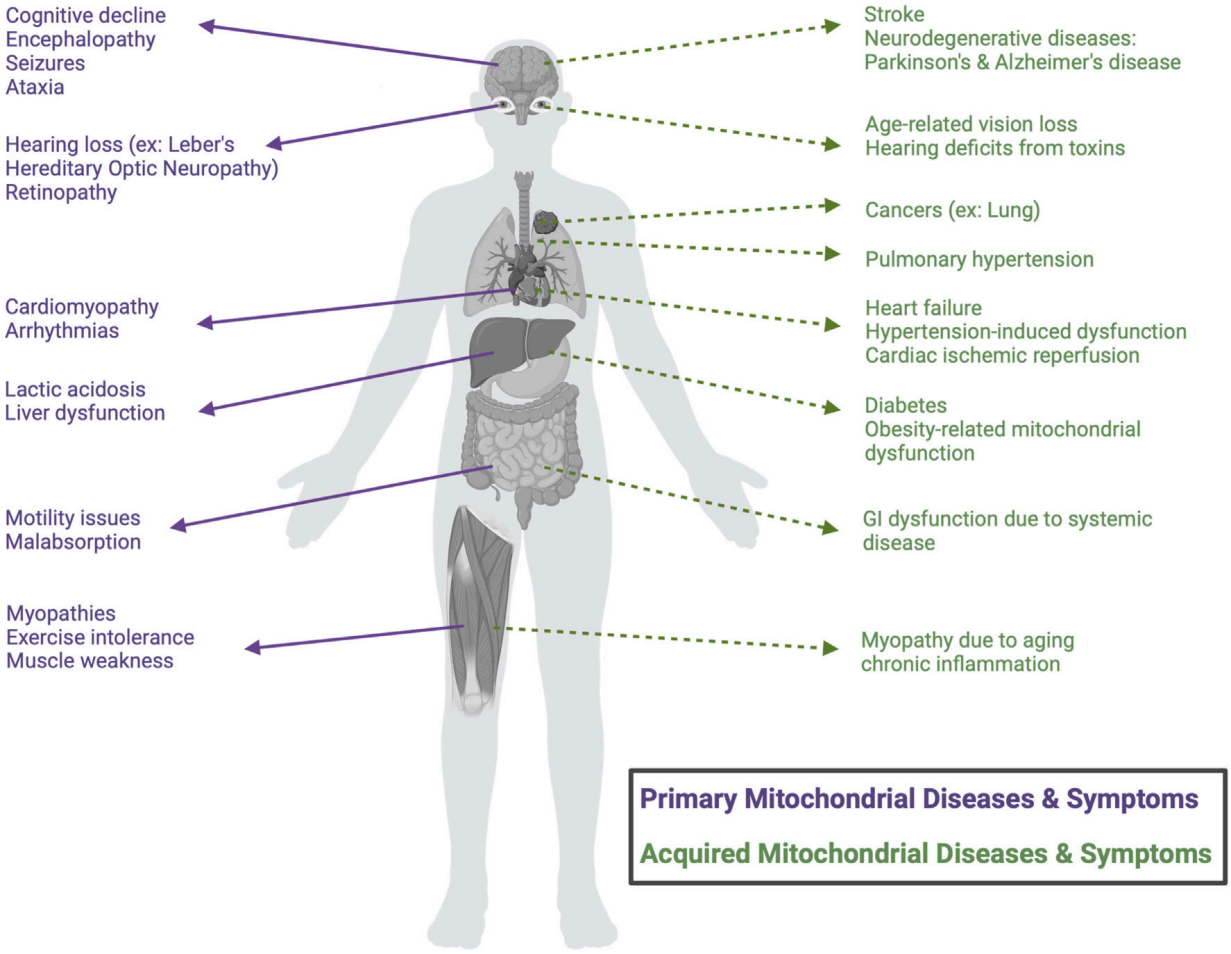
Classification of mitochondrial-driven diseases and symptoms. Schematic classification of the distinction between primary mitochondrial diseases (purple), caused by direct genetic mutations, and mitochondrial dysfunction associated with acquired, complex diseases, which result from other systemic conditions (green). Common clinical manifestations are categorized by organ involvement.

Mitochondrial-driven diseases encompass a diverse group of disorders that can be either maternally inherited or acquired; both are rooted in the dysfunction of mitochondria, the cellular powerhouses responsible for energy production and metabolic regulation (Clemente-Suárez et al., 2023). These diseases may originate from genetic mutations affecting mitochondrial DNA (mtDNA) or nuclear DNA (nDNA) encoding mitochondrial proteins, and/or from acquired factors such as oxidative stress, environmental toxins, or age-related degeneration (Clemente-Suárez et al., 2023; Zong et al., 2024). The human mitochondrial genome contains 37 genes, whereas the nuclear genome encodes approximately 1,100 genes that are associated with the mitochondria. Thus, while all 37 mitochondrial genes have been implicated in primary mitochondrial diseases, nearly eight times the number of nuclear genes (close to 300) have also been linked, highlighting the imbalance in location of mutations (Chinnery and Hudson, 2013; Mccormick et al., 2018). Broadly, mitochondrial-driven diseases can be classified into two categories: i) primary mitochondrial diseases, which stem from direct genetic mutations of mtDNA or nDNA encoding for proteins expressed in the mitochondria and ii) mitochondrial dysfunction associated with complex diseases, observed in systemic conditions such as diabetes (Yoon et al., 2011 February 1), ischemia reperfusion injury (Archer, 2013), neurodegenerative diseases (Jiang et al., 2015 July 29), pulmonary hypertension (Archer et al., 2010 May 11), and cancer (Boland et al., 2013 December 2) - where the causal relationship between mitochondrial dysfunction and disease pathogenesis remains an area of investigation (referred to herein as “acquired mitochondrial diseases”, Figure 1).

The clinical spectrum of these disorders is highly variable, reflecting the diverse roles of mitochondria in energy-intensive tissues such as the brain (Stroke, 2024), heart (Meyers et al., 2013), and skeletal muscles (Chen et al., 2022). Epidemiological studies suggest that primary mitochondrial-driven diseases affect approximately 1 in 5,000 individuals worldwide, making them one of the most common groups of genetic disorders (Parikh et al., 2015; Ng and Turnbull, 2016; Buajitti et al., 2022). Diagnosis and treatment of both primary and secondary acquired mitochondrial diseases remain significant challenges, as all of these conditions often present with heterogeneous symptoms and are thus challenging to diagnose, and for most, no curative therapies are available. Existing therapeutic strategies focus on either a ‘one-size-fits-all’ approach, incorporating symptom management through diet, exercise, and antioxidants, or precision medicine, which leverages patient-specific (genetic) insights to tailor therapeutic interventions, which are also largely focused on prevention of symptom progression (Parikh et al., 2009; Muraresku et al., 2018; Bottani et al., 2020).

Nuclear DNA also plays a crucial role in modulating mitochondrial function by encoding the majority of essential mitochondrial proteins, including many involved in oxidative phosphorylation, mitochondrial dynamics, and mitochondrial quality control mechanisms (Ali et al., 2019). While nDNA does not directly alter mtDNA transcription, it regulates mtDNA maintenance and expression through nuclear-encoded factors (Ali et al., 2019). Mutations in these nuclear genes can also lead to secondary instability of the mitochondrial genome, resulting in mtDNA depletion or deletions, which in turn cause later-onset primary mitochondrial disease (Rusecka et al., 2018). Mutations or epigenetic changes in nuclear-encoded genes (e.g., SOD2) can also directly cause acquired mitochondrial mutations or alter mitochondrial function, leading to oxidative stress and secondary mtDNA damage, demonstrating that the antioxidant–ROS link between the nuclear and mitochondrial genomes is a major mechanism of mtDNA damage (Miao and Clair, 2009). In cases of mtDNA dysfunction, nDNA can upregulate compensatory pathways such as mitophagy and antioxidant defenses to mitigate cellular stress (Ali et al., 2019). However, the effects of nDNA are not always beneficial; incompatibilities between nuclear and mitochondrial genomes can exacerbate disease phenotypes by disrupting coordinated gene expression and impairing mitochondrial function (Ma et al., 2016). Understanding these interactions is critical for developing therapies that target both nuclear and mitochondrial contributions to disease pathology.

### 1.2 Modelling mitochondrial-driven diseases

Preclinical models have provided fundamental insights into the defining features and mechanisms of mitochondrial-driven diseases. Many of these disorders are characterized by dysfunction of the electron transport chain (ETC) causing impaired oxidative phosphorylation (OXPHOS), excessive production of reactive oxygen species (ROS), and abnormal mitochondrial dynamics (fission, fusion, and mitophagy) (Ng and Turnbull, 2016; Bhatti et al., 2017; Chen et al., 2023; Choi et al., 2024). The phenotypic variability observed across acquired mitochondrial diseases, including neurodegeneration (Parkinsonism and other neurodegenerative diseases) (Kanungo et al., 2018; Gropman et al., 2024), cancer (Wen et al., 2025), cardiomyopathies, pulmonary hypertension (Mocumbi et al., 2024), ischemia reperfusion injury (Sharp et al., 2014) and metabolic syndromes (obesity, hyperglycemia, and a range of liver problems) (Dunham-Snary and Ballinger, 2013), underscores the clinical heterogeneity that complicates diagnosis and management. Additionally, primary mitochondrial diseases are also highly variable in terms of the manifestations in various organ systems (ranging from single to multi-organ system targets), ii) predisposed population (often ranging from infancy to adulthood), iii) symptom severity (spanning mild to fatal), and iv) natural history of the disease (with some patients experiencing prolonged periods of stability followed by rapid decline) (Muraresku et al., 2018; Klopstock et al., 2021).

Emerging precision medicine strategies have enhanced diagnostic accuracy, but significant challenges remain in identifying effective treatment options, particularly for primary mitochondrial diseases. This is partly due to the absence of reliable disease-modifying therapies and limited preclinical models that isolate the contributions of mtDNA to the disease signs, symptoms and outcomes. Preclinical models have been instrumental in elucidating mitochondrial dysfunction and its role in disease. Over the past five decades, research has advanced the study of mtDNA-specific effects in both primary and acquired mitochondrial diseases and have supported the translation of novel technologies into clinical applications. These models are essential for investigating mitochondrial biology, assessing the functional impact of genetic mutations, facilitating exploration of multi-factorial pathologies resulting from underlying mitochondrial dysfunction, and evaluating potential therapeutic strategies. Their development has evolved alongside advancements in molecular biology and bioengineering, allowing researchers to replicate and “isolate” mitochondrial pathophysiology with increasing accuracy, employing various techniques and breeding strategies to ‘switch’ mitochondrial genetic background in cells and animals. Each method presents distinct advantages and limitations in terms of clinical applicability.

*In vitro* models, including “cybrid” cell lines (cytoplasmic hybrid cells produced by combining a cell with independent mitochondrial and nuclear donors), and induced pluripotent stem cells, provide a controlled environment for studying mitochondrial dysfunction at the cellular level and enable high-throughput screening of putative therapeutic compounds (Vaz et al., 2024). In contrast, *in vivo* models, such as genetically modified mice and zebrafish, allow for the investigation of both systemic and tissue-specific consequences of mitochondrial dysfunction (Kesterson et al., 2016; Otsuka and Matsui, 2023; Welch et al., 2023). *In vitro* and *in vivo* models are complementary: *in vitro* systems excel in informing mechanistic research, while *in vivo* models offer insights into the role of mitochondria in disease pathogenesis and allow testing of therapeutic agents. Recent advancements, such as development of organoid-based models and mitochondrial replacement therapy are being explored for their potential to treat both primary and acquired mitochondrial diseases, by restoring healthy organelle function. Future research aims to refine mitochondrial genome editing as a curative strategy for primary mitochondrial diseases.

This review synthesizes current knowledge on preclinical models of mitochondrial-driven diseases, evaluating their scientific utility and translational potential. We will explore how manipulating mitochondrial genetics, gene mutations, and mtDNA/nDNA mismatch impacts the fidelity of preclinical disease modelling. We review models that were initially developed to study primary pathologies but evolved for use in investigation of secondary or acquired mitochondrial diseases. Key themes in this review include: (i) an overview of existing preclinical models used in mitochondrial research, (ii) a comparative analysis of strengths and limitations of *in vitro* and *in vivo* systems, and (iii) emerging innovations in model development. By critically examining the evolution of preclinical models, this review identifies methodological gaps and proposes future directions to enhance their impact. Collectively, this work underscores the essential role of preclinical research in bridging basic mitochondrial biology and clinical application, advancing efforts to develop effective treatments for mitochondrial-driven diseases.

### 1.3 Evolution and development of preclinical models of mitochondrial-driven diseases

**FIGURE 2:**
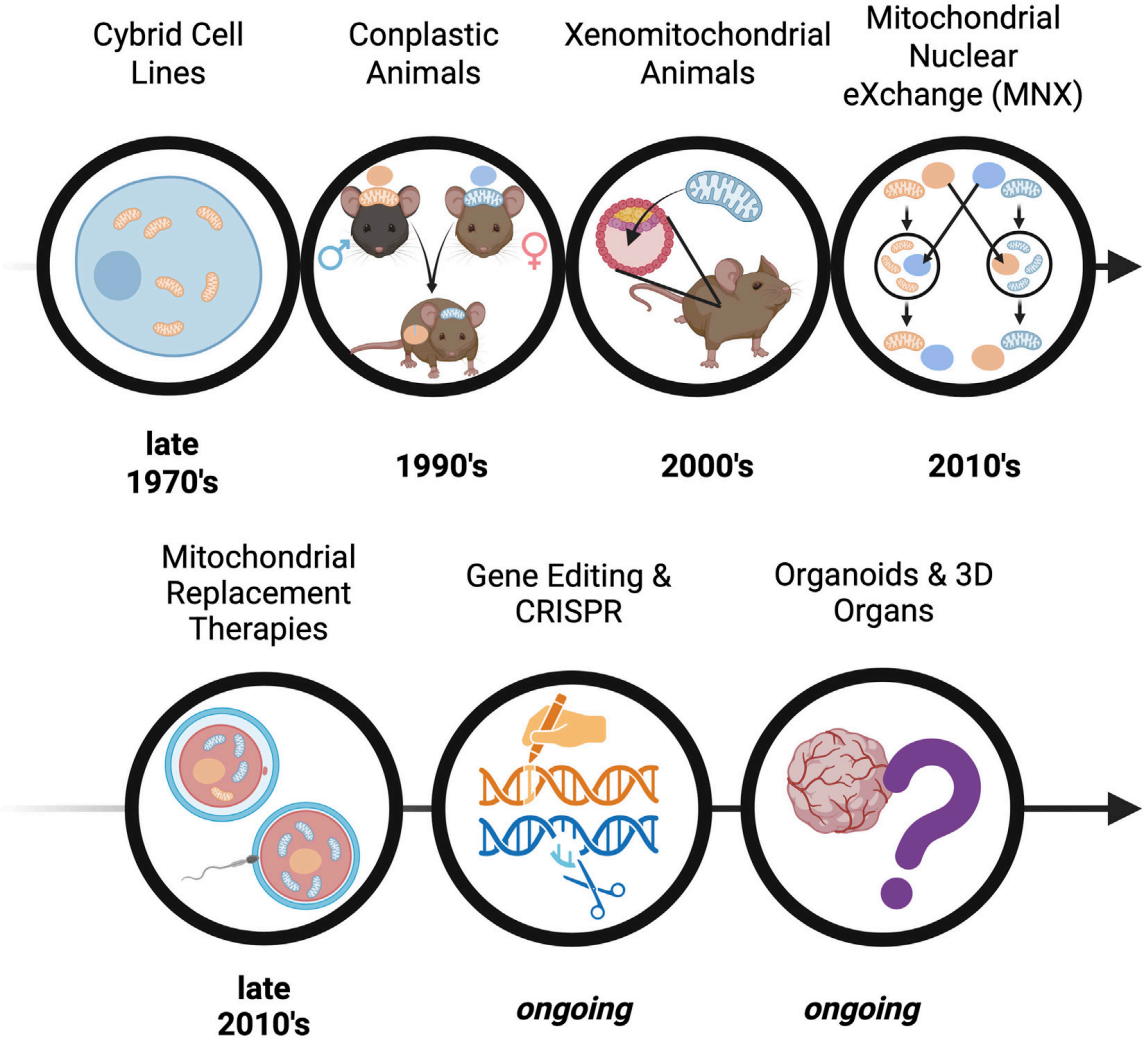
Timeline of discovery - The evolution of pre-clinical models for studying mitochondrial-driven diseases. The progression of mtDNA-based models is depicted, starting with early in vitro modelling with cybrid cell lines (cytoplasmic hybrid cells produced by combining a cell with independent mitochondrial and nuclear donors), progressing to in vivo models such as: conplastic animals (inbred strains possessing identical nuclear genomes but differing mtDNA), xenomitochondrial animals (integration of foreign mtDNA through cybrid cell lines), and Mitochondrial Nuclear eXchange (reciprocally exchanged pronuclei). Advancements in translational techniques are also depicted, including mitochondrial replacement therapy and CRISPR-based gene editing; these approaches reflect increasing sophistication in modelling mitochondrial dysfunction and advancing therapeutic strategies.

The development of preclinical models for mitochondrial-driven diseases has undergone significant evolution, marked by key milestones (Figure 2) that have shaped the field, such as: i) the development of technologies to study mtDNA mutations in a controlled nuclear background, enabling the functional analysis of the consequences of mitochondrial defects, ii) the successful integration of mitochondria from one species into another, allowing the study of the mitochondrial abnormalities to disease progression, iii) the creation of models where mtDNA from one strain is introduced into the nuclear background of another through breeding, helping dissect mtDNA-nDNA interactions and consequences of mismatch, and lastly (iv) the development of a model that reciprocally exchanges nuclei between embryos with different mtDNA backgrounds, revealing the independent effects of mtDNA while controlling the nDNA background. Early studies relied heavily on yeast and bacterial systems to explore mitochondrial biogenesis and function (Feuermann et al., 2003; Meunier et al., 2013). Subsequently genetic engineering facilitated the creation of mammalian models with targeted mtDNA or nDNA mutations (Lin et al., 2012; Kesterson et al., 2016; Gammage et al., 2018b; Welch et al., 2023), enabling increasingly sophisticated analyses of mitochondrial dysfunction and its systemic consequences—topics explored in the following sections.

Both of these methods can introduce undesired effects, such as mtDNA heteroplasmy, the presence of more than one type of mitochondrial genome (in xenomitochondrial animals) and/or heteroplasmic nDNA (as a product of back-crossing animal strains with distinct nDNAs). To address these model limitations, the Mitochondrial Nuclear eXchange (MNX) mouse model was created. These mice possess reciprocally exchanged pronuclei between mouse strains; the MNX procedure ensures purity and homogeneity of the desired nDNA and mtDNA backgrounds. For example, MNX mice have been created using C57BL6/J and C3H/HeN mice to explore obesity and metabolic syndrome (Dunham-Snary et al., 2018), while FVB/NJ, C57BL/6J, and BALB/cJ MNX mice have been used to investigate progression and severity of breast cancer (Feeley et al., 2015).

#### 1.3.1 Cytoplasmic hybrid (“cybrid”) cell lines

**FIGURE 3:**
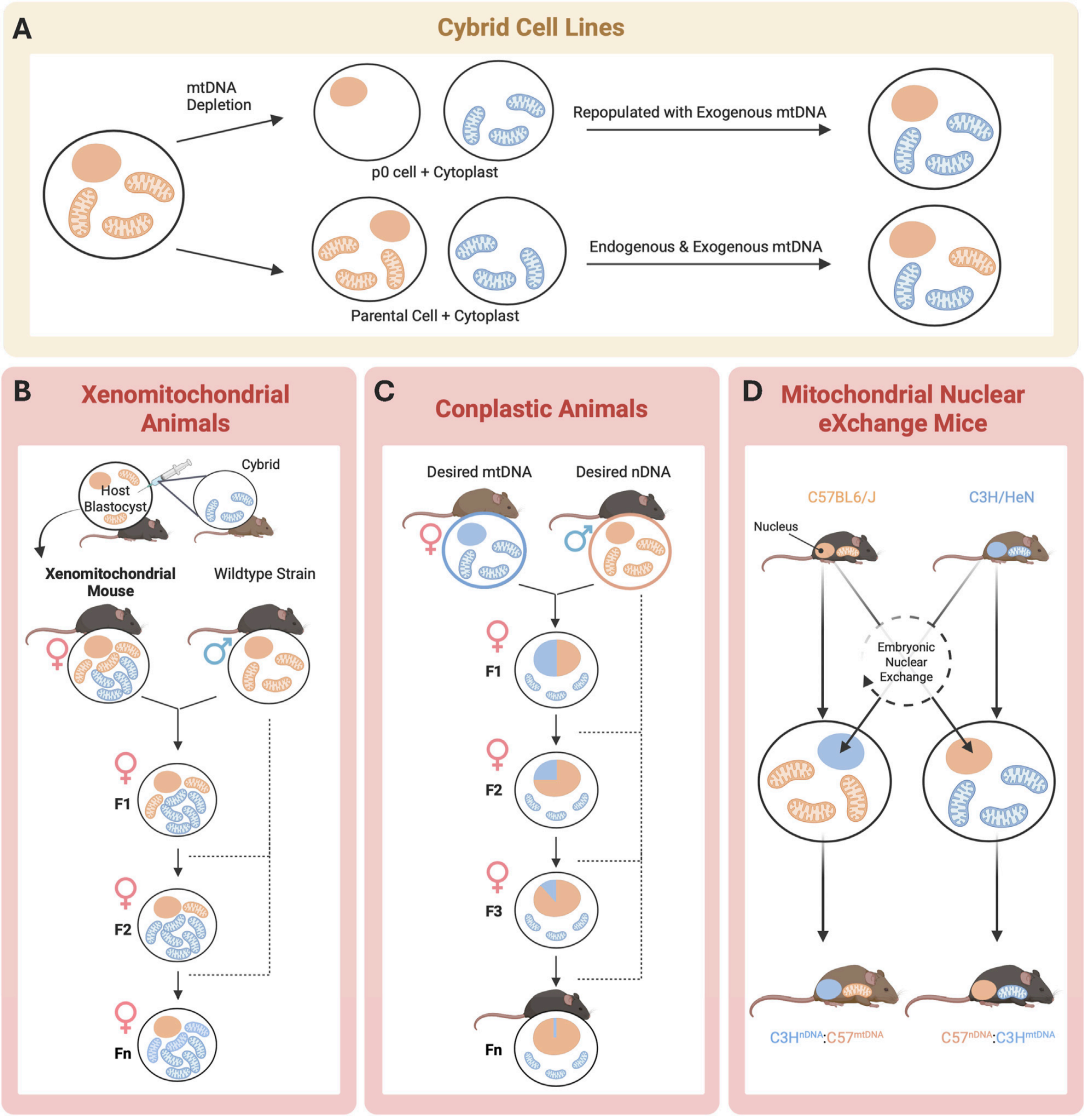
Schematic of mtDNA model generation. *In vitro* (yellow) and in vivo approaches (pink) to achieve desired nuclear DNA (orange) and mtDNA (blue). **(A)** Cybrid cell lines offer the most precise method for studying mtDNA effects but lack whole-body system integration; **(B)** Xenomitochondrial animals enable the study of mtDNA-nDNA interactions and whole- body disease phenotypes but require extensive backcrossing and have a high risk of heteroplasmy; **(C)** Conplastic animals enable the study of mtDNA-nDNA interactions but are expensive to breed and maintain, with heteroplasmy still posing a challenge; and **(D)** Mitochondrial Nuclear eXchange Mice eliminate heteroplasmy and provide full genetic control, but high cost and maintenance requirements limit use (**(D)** adapted from Shastry et al., 2025).

First derived in 1975 by Wallace, Bunn, and Eisenstadt, cytoplasmic hybrid (“cybrid”) cell lines are generated by the fusing of nucleated cells that have had their endogenous mtDNA depleted via exposure to ethidium bromide with enucleated cells (“cytoplasts”), which harbor the mtDNA signature of interest (Figure 3A; Wilkins et al., 2014). Since the nuclear background of different cell lines can be kept constant in cybrid cells, this technique allows investigators to study the independent influence of the newly introduced mtDNA on cell functions, such as OXPHOS (Trounce and Pinkert, 2007). Cybrid cell line approaches have been successfully applied to mouse models to study both primary and acquired mitochondrial diseases. Using cybrid cells as an intermediate carrier of mtDNA, embryonic stem (ES) cell cybrids have also been produced in several laboratories (Lyu et al., 2023). Additionally, both homoplasmic and heteroplasmic mice have been produced using cybrid cells as a precursor, allowing for the study of mtDNA inheritance and modeling the effects of mtDNA mutations through the mouse germline (Figure 2; Trounce and Pinkert, 2007).

Cybrid models have significantly advanced our understanding of mitochondrial-driven diseases; for example, studies in the late 1990s of the A3243G mtDNA mutation, associated with mitochondrial encephalopathy, lactic acidosis, and stroke-like episodes (MELAS) syndrome, provided valuable insights into the biochemical thresholds required for dysfunction. Using cybrids generated from cell lines lacking mtDNA (ρ0 cell lines), researchers found that a high mutational burden was necessary to produce biochemical consequences, which were primarily due to impaired translation of a mtDNA-encoded structural protein mTERF (King et al., 1992; Koga et al., 1995; Shoubridge, 1995). Subsequent work by Holt et al. demonstrated that the nuclear background can significantly influence these effects. In 206.3243 cybrid cells, a 95% mutational burden led to an ∼75% reduction in cytochrome c oxidase activity, whereas a lower (85%) mutation burden in a different nuclear background (i.e., B2.3243) caused greater reduction (∼85%) in activity. This indicates that cell lines exhibit differing, cell type-specific, susceptibilities to the mutation (Dunbar et al., 1995). Nuclear-dependent variability in the consequences of a mtDNA mutation has also been observed in studies of Leber’s hereditary optic neuropathy (LHON), a disorder associated with mutations in mtDNA-encoded subunits of ETC Complex I, NADH dehydrogenase. Cybrid experiments beginning in the late 1990s confirmed that the G11778A mutation in *mt-ND4* leads to Complex I defects and impaired ATP synthesis, with the magnitude of these deficiencies differing depending on the nuclear genetic background (Vergani et al., 1995; Hofhaus et al., 1996). Similarly, studies by Wilkins et al. in 2014 employed cybrid models to investigate Parkinson’s disease (Wilkins et al., 2014), one of the first uses of cybrid cells in acquired mitochondrial disease research. By transferring mitochondria from Parkinson’s patients into mtDNA-depleted cells that were otherwise healthy, researchers identified defects in the electron transport chain and heightened oxidative stress, underscoring the role of mtDNA alterations in Parkinson’s disease (Miller et al., 1996; Swerdlow et al., 1996; Gu et al., 1998). These foundational studies underscored the importance of nuclear-mitochondrial interactions in determining mitochondrial dysfunction severity and disease expression in both primary and acquired mitochondrial diseases.

In recent years, cybrid models have continued to shed light on mitochondrial dysfunction in various pathologies, including drug-induced liver disease. A study in 2023 by Ball et al. used HepG2-derived transmitochondrial cybrids to evaluate how mtDNA variations affect mitochondrial function and vulnerability to drug-induced liver injury (Ball et al., 2023). The findings indicated that specific haplogroups (populations sharing similar mtDNA polymorphisms) are more susceptible to inhibition by hepatotoxic drugs, such as flutamide, 2-hydroxyflutamide, and tolcapone, as evidenced by the haplogroup heterogeneity in the magnitude of uncoupling of the respiratory chain and alteration of mitochondrial Complexes I and II function (Ball et al., 2023). These results indicate that specific mtDNA haplogroups respond differently to mitochondrial toxicants, emphasizing the key role of mtDNA background in drug-induced mitochondrial dysfunction (Ball et al., 2023). Cybrid models offer a versatile *in vitro* approach to dissect these complex mito-nuclear interactions, providing valuable insights into disease mechanisms and potential therapeutic targets.

#### 1.3.2 Xenomitochondrial and conplastic mouse strains

To better capture the systemic and tissue-specific manifestations of mitochondrial-driven diseases, researchers have extended cellular-level findings from cybrid cell lines into whole-animal systems. Xenomitochondrial mice are a useful model for studying disease progression at the tissue and organismal levels (Figure 2). They are generated via integration of foreign mtDNA through cybrid cell lines, and can be created through two methods using ES cells (Cannon et al., 2011). Mice are then bred for multiple generations via backcrossing to produce progeny harboring the mtDNA of interest (Figure 3B; Cannon et al., 2011). Conplastic animals, like xenomitochondrial animals, are inbred strains possessing identical nuclear genomes but differing mtDNA (Figure 2; Pravenec et al., 2021). Unlike xenomitochondrial models that use direct cybrid integration and subsequent back-crossing, conplastic animals rely solely on selective breeding (Figure 3C). Both conplastic and xenomitochondrial animal breeding require backcrossing for ∼ 10 generations to ensure nuclear and mitochondrial genomes remain nearly identical (∼99.8%) to the recipient strain (Sligh et al., 2000). Unlike cybrid cell lines, conplastic and xenomitochondrial models allow for the study of mitochondrial-driven disease progression at multiple biological scales, ranging from cellular dysfunction to organ function and systemic pathology *in vivo*.

Landmark studies using xenomitochondrial mice have specifically investigated the consequences of mitochondrial-nuclear genetic mismatches. For example, in 2004, McKenzie et al. developed xenomitochondrial mice by introducing *Mus terricolor* (light brown) mitochondria into *Mus musculus domesticus* (dark brown). Despite significant evolutionary divergence, the offspring exhibited normal development and physiology (Mckenzie et al., 2004). Conplastic mouse models offer additional insights into how nuclear-mtDNA variations influence disease phenotypes (Gammage et al., 2018b). These models involve selecting a female with the desired mitochondrial background and breeding them with a male of the desired nuclear background. Continuous backcrossing of female progeny to males of the desired nDNA reinforces the desired nuclear genetic lineage while maintaining the ‘mismatched’ mtDNA lineage (which is exclusively maternal in origin). For instance, in 2012, Sharpley et al. used conplastic mice to demonstrate that mtDNA can influence cognitive function and anxiety-like behavior (Sharpley et al., 2012). Specifically, using NZB mice and 129S6 mtDNA background in the presence of a C57BL/6J nDNA background, they found that mice with mismatched nDNA:mtDNA exhibited accentuated stress response and cognitive impairment, highlighting the importance of mitochondrial-nuclear interactions in neurological phenotypes associated with mitochondrial-driven diseases (Sharpley et al., 2012).

Recent research using xenomitochondrial and conplastic mouse models continues to reveal the complexities of mitochondrial-driven diseases. For example, in 2020, Lopez Sanchez et al. investigated the nuclear response to divergent mitochondrial DNA genotypes using xenomitochondrial mice (Lopez Sanchez et al., 2020). They introduced mtDNA from *Mus Spretus, M. terricolor, Mus caroli, or Mus Pahari* into nDNA of *M. musculus domesticus* (Lopez Sanchez et al., 2020). The results indicated that baseline interferon gene expression was lower in xenomitochondrial mice relative to controls, leading to a reduced interferon-dependent innate immune response and viral control when challenged with pathogens, such as with herpes simplex virus (Lopez Sanchez et al., 2020). Their findings indicated that nuclear-mitochondrial interactions modulate the interferon immune response, providing insights into the role of mitochondrial-nuclear crosstalk in immune regulation (Lopez Sanchez et al., 2020). Comparably, a study by Scotece et al. in 2021 employed a conplastic mouse model to assess the impact of mtDNA variations on joint aging (Scotece et al., 2021). They developed a conplastic strain with the C57BL/6JOlaHsd nuclear genome and NZB/OlaHsd mtDNA and found that these mice exhibited reduced joint damage and lower expression of senescence markers compared to controls, suggesting a protective effect of certain mtDNA haplotypes against age-related osteoarthritis (Scotece et al., 2021). This work is supported by observations across species that mitochondrial haplotype can significantly influence processes from biologic aging (Nguyen et al., 2025), to disease susceptibility (Dunham-Snary et al., 2018; Sammy et al., 2021).

These recent developments highlight the ongoing utility of xenomitochondrial and conplastic mouse models in unraveling the genetic and molecular underpinnings of mitochondrial-driven diseases, understanding the mitochondrial contribution to complex diseases, and exploring novel treatment strategies (Mckenzie et al., 2004; Sharpley et al., 2012; Wilkins et al., 2014; Lopez Sanchez et al., 2020; Scotece et al., 2021). However, both xenomitochondrial and conplastic approaches can introduce undesired effects, such as mtDNA heteroplasmy in xenomitochondrial animals. This limits the ability to correctly attribute an observed biologic effect to a specific mitochondrial genotype (or mito-nuclear interaction). Another undesired effect is selection for heteroplasmic nuclear DNA (as a product of back-crossing animal strains with distinct nDNAs) in conplastic strains. These issues are relatively common in both xenomitochondrial and conplastic models and have been widely documented (Wallace and Chalkia, 2013). Their confounding impact can be significant, potentially overshadowing the intended genotype-phenotype relations (Nagao et al., 1998). However, they are generally detectable through targeted molecular assays, although routine checks are necessary to avoid misinterpretation.

#### 1.3.3 Mitochondrial-nuclear eXchange (MNX) mouse models

To address concerns with nuclear and mitochondrial genetic heteroplasmy, the Mitochondrial-Nuclear eXchange (MNX) mouse model was created. Kesterson et al. discusses the generation of colony-founding MNX mice in detail (Figure 2; Kesterson et al., 2016). Briefly, these mice possess reciprocally exchanged *pronuclei* (the combined structure of haploid male and female gametes after fertilization, but prior to fusion of genetic material) between mouse strains, and the MNX procedure ensures 100% of both the desired nDNA and mtDNA backgrounds. The first MNX mice were generated using cardiometabolic disease- (CMD) prone C57BL/6J and CMD-resistant C3H/HeN mice (Figure 3D). CMD is an umbrella term for a constellation of interrelated disorders, including abdominal obesity, vascular disease and hypertension, non-alcoholic fatty liver disease, insulin resistance, dyslipidemia, and high fasting glucose levels (Riediger and Clara, 2011). MNX mice differ from previously discussed models of mtDNA manipulation, as all F1 progeny possess 100% of the desired nuclear and mitochondrial DNA backgrounds, circumventing both the nuclear and mitochondrial genetic heteroplasmy challenges associated with backcrossing. MNX mice exploit the maternal inheritance of mtDNA for colony maintenance by breeding MNX dams with nDNA-matched sires (e.g., a female MNX mouse with C57 nuclear DNA + C3H mitochondrial DNA will be bred with a wild-type C57BL6/J male, and all resultant progenies will also be C57^nDNA^:C3H^mtDNA^ MNX mice, Figure 3D). With this breeding strategy, pronuclear transfer does not need to be performed repeatedly, as long as the female MNX used as colony founders have been screened for mtDNA homoplasmy via tissue sampling.

MNX mouse models have proven invaluable in elucidating the role of mtDNA in various acquired mitochondrial diseases, and recent findings in rats demonstrating the importance of mtDNA haplotype in aging support the use of MNX techniques to exploit these haplotype-specific differences for mechanistic studies. Fetterman et al. used MNX mice with mtDNA exchanged between C57BL/6J and C3H/HeN strains to assess the impact of mtDNA polymorphisms on cardiac function (Fetterman et al., 2013). They discovered that mtDNA from C57BL/6J mice conferred increased susceptibility to cardiac stress, evidenced by higher ROS production, suppressed mitochondrial bioenergetics, and elevated mitochondrial membrane potential, *independent of the nuclear background* (Fetterman et al., 2013). This work underscored the significant influence of mtDNA on bioenergetics and disease susceptibility. Feeley et al. employed the MNX technique to generate mice with an FVB/NJ nuclear background and either C57BL/6J or BALB/cJ mtDNA (Feeley et al., 2015). Using this MNX model, they were able to uncover that mtDNA influences primary tumor latency and metastasis, specifically that C57BL/6J mitochondrial genetic background slowed tumor growth in the classically prone mouse possessing FVB/NJ nDNA, underscoring the significance of mtDNA in cancer progression (Feeley et al., 2015). Dunham-Snary et al. used the MNX mouse model to investigate diet-induced obesity and define how nuclear-mitochondrial interactions affect metabolism (Dunham-Snary et al., 2018). They found that different mtDNA backgrounds influenced whole-body metabolism, adiposity, and gene expression in white adipose tissue, with RNA sequencing revealing significant nuclear gene regulation by mtDNA (Dunham-Snary et al., 2018).

Building upon these foundational studies, recent research has expanded the application of MNX models to explore the nuanced roles of mtDNA in metabolic regulation. Sammy et al. investigated the impact of mtDNA background on glucose metabolism and insulin sensitivity in MNX mice (Sammy et al., 2021). They found that mice with CMD-resistant mtDNA and CMD-prone nDNA exhibited lower adiposity, reduced plasma leptin levels, increased insulin sensitivity (reflected by lower HOMA-IR values), and enhanced muscle insulin signaling, when fed a standard rodent chow diet (Sammy et al., 2021). Additionally, Shastry et al. conducted a tissue and circulating metabolomic analysis in MNX mice fed a high fat diet (Shastry et al., 2025). Their results show that CMD-prone mtDNA increases body fat percentage across both CMD-prone and CMD-resistant nDNA backgrounds. Further, that mtDNA variation influences levels of circulating metabolites linked to incomplete beta oxidation, suggesting a mechanism by which mtDNA contributes to insulin resistance (Shastry et al., 2025). Sammy et al. and Shastry et al. demonstrated the protective effects of CMD-resistant mtDNA and harmful effects of CMD-prone mtDNA, respectively. These findings shed light on a putative mechanism by which mtDNA exerts its effects on varying nuclear genetic backgrounds. This idea was first proposed by Kopinski et al., who used cybrid cell lines to demonstrate that increased mtDNA heteroplasmy was associated with changes to post-translational modifications of histone proteins, thereby influencing the epigenetic regulation of nuclear gene expression (Kopinski et al., 2019).

Beyond the insights gained from studies of metabolism and cancer, the versatility of MNX models has enabled researchers to explore new nDNA-mtDNA combinations to elucidate complex disease mechanisms. For instance, in 2023 Zou et al. leveraged the MNX mouse model to investigate the role of mtDNA in autoimmune type 1 diabetes (Zou et al., 2023), a novel application of this technology. By combining nuclear DNA from diabetes-prone NOD/ShiLtJ mice with mtDNA from non-diabetic C57BL/6J mice, they observed improved survival and delayed diabetes onset in the MNX mice (Zou et al., 2023). This improvement was linked to normalized activity of respiratory complex I in mice with C57BL/6J mitochondria, highlighting the critical influence of mtDNA on the disease phenotype (Zou et al., 2023). These collective findings underscore the unique capacity of MNX models to dissect the complex interplay between mitochondrial and nuclear genomes in disease, paving the way for targeted therapeutic strategies aimed at modulating mitochondrial function.

### 1.4 Limitations of existing models

While preclinical models have proven instrumental in dissecting mitochondrial-driven diseases, it is crucial to acknowledge the inherent strengths and limitations that accompany each approach. *In vitro* systems like cybrid cell lines offer accessibility and scalability for mechanistic studies yet often fail short in replicating the intricate systemic interactions between nDNA and mtDNA found *in vivo*. Conversely, animal models offer a more comprehensive representation of mitochondrial dysfunction within a biological system, but each presents unique challenges related to replication of human pathophysiology.

While Table 1 provides a broad overview, it is important to consider specific strengths and limitations within each model system. For instance, cybrid cell lines have been extensively utilized to investigate mitochondrial dysfunction at a cellular level, particularly in the context of acquired mitochondrial diseases such as Parkinson’s disease (Trimmer and Bennett, 2009), HIV (Lu et al., 2013), human herpes virus type 8 (Gustafson et al., 2000), and primary mitochondrial diseases such as MELAS syndrome (Koga et al., 1995), LHON (Jun et al., 1996), and Leigh syndrome (Trounce et al., 1994). The general consensus from studies employing cybrid cell lines is that reductions in respiratory chain complex activity, oxygen consumption, and ATP synthesis can be effectively modeled, making cybrids a valuable tool for studying certain aspects of mitochondrial dysfunction (Sazonova et al., 2018). However, a key limitation remains: cybrid cell lines are unable to fully recapitulate the complex systemic interactions necessary to model disease progression at the tissue or organismal level, simply because they are cells and not animals. This inherent constraint spurred to transition to animal-based models, which offer the potential to elucidate mtDNA genotype-phenotype correlations in a more physiologically relevant context.

**TABLE 1:** Comparison of *in vivo* and *in vitro* preclinical mtDNA models.

mtDNA Model	Strengths	Limitations
Cybrid Cell Lines	• Most precise method for isolating mtDNA effects • Rapid and scalable technique • Relatively inexpensive	• Lacks system integration • Unable to model disease progression
Xenomitochondrial Animals	• Allows study of mtDNA-nDNA interactions • Can mimic disease phenotype and progression • Representative of whole-body phenotype	• Requires extensive backcrossing • High risk of heteroplasmy
Conplastic Animals	• Allows study of mtDNA-nDNA interactions • Can mimic disease phenotype and progression • Representative of whole-body phenotype	• Requires extensive backcrossing • Risk of heteroplasmy • Expensive to breed and maintain
Mitochondrial Nuclear eXchange Mice	• Control over mtDNA-nDNA interactions • Achieve 100% of targeted nDNA and mt DNA genomes immediately • Models’ disease at the organismal level • Avoids backcrossing and heteroplasmy entirely	• Requires extensive colony maintenance • Expensive to obtain and maintain • Patented techniques

Models are compared in terms of their strengths and limitations for studying mitochondrial diseases. *In vitro* models (highlighted in yellow boxes) and *in vivo* models (highlighted in pink boxes). This figure highlights the unique contributions of both model types to mitochondrial research while also addressing their respective limitations, emphasizing the complementary roles these models play in advancing our understanding of mitochondrial dysfunction.

While animal-based models offer improved systemic relevance, they too possess limitations that must be considered. One notable challenge with xenomitochondrial and conplastic mtDNA models lies in the potential for heteroplasmy–the intercellular coexistence of wild-type and mutant (nDNA and/or mtDNA) strands. As highlighted in a study by Hirose et al. this phenomenon can significantly impact a wide spectrum of diseases and physiological processes (Hirose et al., 2018). Specifically, their conplastic mouse strain, generated to carry the mtDNA of an AKR/J mouse strain in a C57BL/6J nuclear genomic background, exhibited >20% heteroplasmy. These conplastic mice demonstrated a dysregulation of multiple metabolic pathways, resulting in impaired glucose metabolism compared to wild-type mice with lower levels of heteroplasmy. These results indicated that reduced heteroplasmy leads to greater upregulation of mtDNA translation and that levels of heteroplasmy correlate to mtDNA copy number, glucose metabolism, and mitochondrial functions when normalized to mtDNA:nDNA copy number. Taken together, these findings suggest that physiologically relevant differences in mtDNA heteroplasmy impair metabolic health and lifespan in mice (Hirose et al., 2018). Critically, these models, which rely on inbreeding and backcrossing to manipulate mtDNA, are often unable to product full homoplasmy, complicating the interpretation of results.

While xenomitochondrial and conplastic models provide valuable insights, the extrapolation of findings from animal models to human conditions remains a significant hurdle. Species-specific differences in physiology, including, but not limited to mitochondrial function, present ongoing challenges in translating results directly. This difficulty in translation extends to the MNX mouse model, where, although homoplasmy is achieved, species-specific differences must still be considered. Additionally, a limitation to the MNX mouse model is that their use requires access to patented technology, which can restrict availability to researchers. This can increase costs, limit collaboration opportunities, and slow the integration of MNX models for studying secondary acquired mitochondrial diseases.

### 1.5 Lessons from rodent models in mitochondrial research

Despite the inherent limitations of rodent models, they have been invaluable in mitochondrial research, paving the way for innovative therapeutic strategies. Specifically, xenomitochondrial mice represent the first viable trans-mitochondrial mice with intended homoplasmic replacement of endogenous mtDNA, confirming the ability to produce (or correct) mitochondrial defects in mice through the xenomitochondrial approach (Mckenzie et al., 2004). There is a range of naturally occurring evolutionary divergence in mitochondrial functions within the rodent subfamily that can be used to create different rodent lines with a range of OXPHOS defects, as described by Singh et al. (2022). For example, there are xenomitochondrial mouse models with myopathy, dilated cardiomyopathy, and perinatal or *in-utero* lethality (Lyu et al., 2023). Additionally, there are xenomitochondrial models that can replicate and transcribe foreign mtDNA, potentially bypassing defective endogenous mitochondria, as seen in mitochondrial transfer therapies for neurodegenerative diseases (Bustamante-Barrientos et al., 2023).

At the same time that the Mitochondrial-Nuclear eXchange mouse model was being developed, a similar technique was investigated in non-human primates using maternal spindle transfer (replacing the cytoplasm of an oocyte with that of a donor), rather than pronuclear transfer (Tachibana et al., 2009). Tachibana et al. reported successful spindle replacement and mitochondrial genome transplantation in rhesus macaques (Tachibana et al., 2009). Like pronuclear transfer in MNX mice, the oocytes that underwent maternal spindle transfer were capable of being fertilized, developing into normal embryos, and producing healthy offspring. Subsequent genetic analysis was able to confirm that nDNA in the infants born originated from the spindle donors, whereas mtDNA came from the cytoplast donor. These results suggest that both ‘MNX approaches’ (pronuclear transfer and maternal spindle transfer), may be able to offer an option to human patients with primary mitochondrial disease, preventing transmission to their offspring (Tachibana et al., 2009) (discussed in detail in Section 3.2).

## 2 Innovations in modeling primary mitochondrial diseases

### 2.1 Advances in genetic and genomic tools

The introduction of sophisticated genetic and genomic tools has significantly transformed preclinical modeling, enabling researchers to dissect primary mitochondrial diseases with increased precision. Techniques such as next-generation sequencing (NGS), single-cell RNA sequencing (scRNA-seq), and epigenomic profiling have revealed the genetic and molecular landscapes of mitochondrial dysfunction (Mahmud et al., 2022; Awuah et al., 2023). These innovations have not only accelerated the identification of pathogenic variants but also facilitated the creation of disease-specific models tailored to mimic human pathologies. For instance, the use of NGS in characterizing mtDNA mutations has provided crucial insights into the genetic basis of MELAS (Grigalionienė et al., 2023). It has also uncovered co-occurring mtDNA variants and tracked somatic mutations that contribute to disease progression, enhancing diagnostic accuracy and patient stratification in MELAS and IgA nephropathy (Choi et al., 2008; Douglas et al., 2014). Similarly, scRNA-seq has been instrumental in uncovering cell-specific transcriptional changes associated with mitochondrial dysfunction, offering new avenues for targeted therapeutic interventions (Lareau et al., 2022; Awuah et al., 2023).

### 2.2 Mitochondrial replacement therapy (MRT)

**FIGURE 4:**
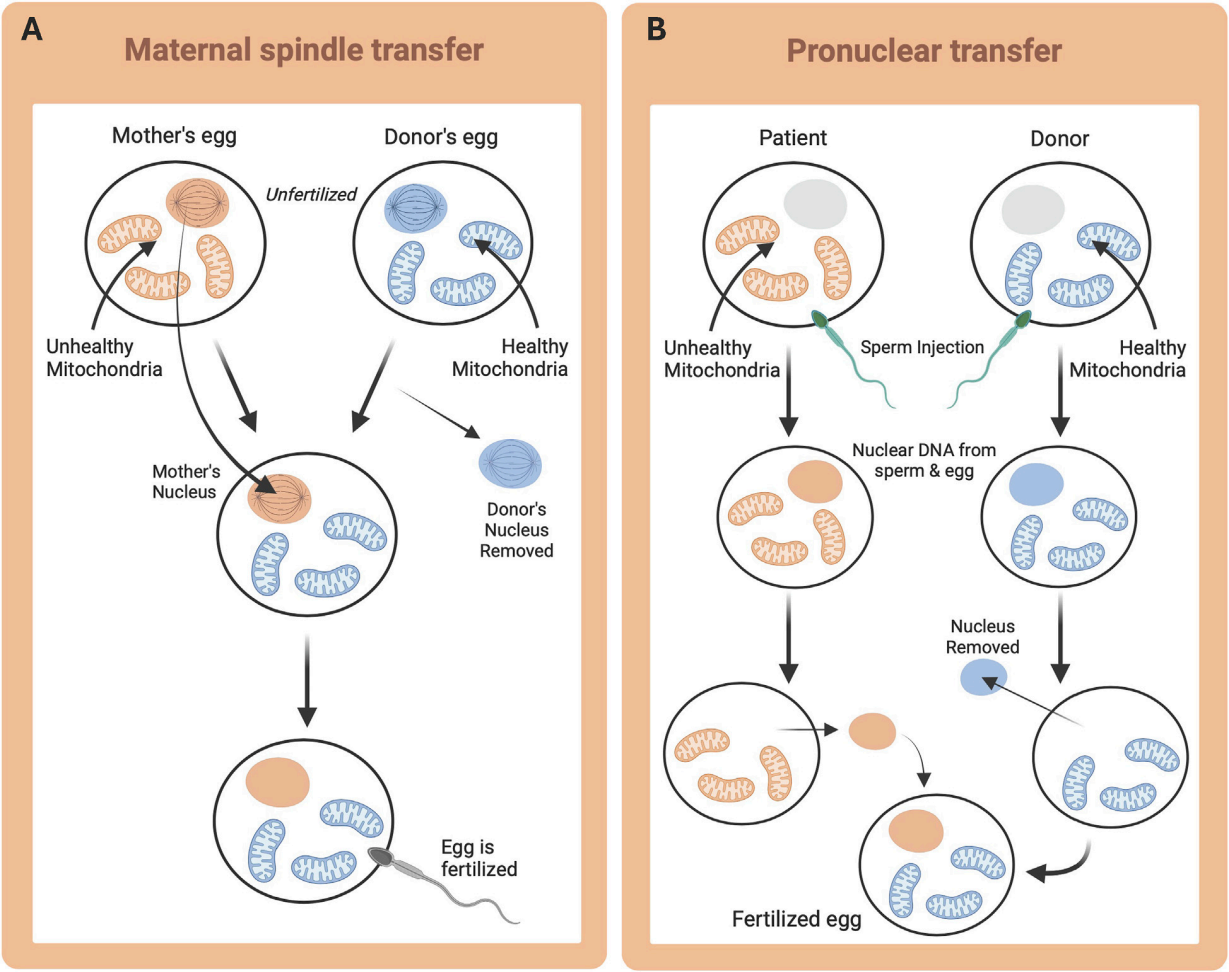
Mitochondrial Replacement Therapy techniques. Desired nuclear DNA (orange) and desired mtDNA (blue). **(A)** Maternal (or Mitotic) spindle transfer, whereby metaphase is triggered in the oocyte, and the spindle is removed and transplanted to a donor oocyte prior to fertilization; and **(B)** Pronuclear transfer, whereby fertilization is achieved *in vitro* and subsequently the pronucleus is removed and transplanted.

Both pronuclear transfer and maternal spindle transfer have been proposed for human mtDNA replacement therapies to ‘treat’ primary mitochondrial diseases by preventing their transmission. The key difference between these two techniques is timing. Maternal spindle transfer occurs before fertilization and thus, prior to the fusion of nuclear genetic material from sperm and egg (Figure 4A; Reznichenko et al., 2016). Alternatively, pronuclear transfer occurs after fertilization, and therefore, any nuclear gene selection influenced by the mitochondria occurs in the presence of the diseased mitochondria (Figure 4B). Maternal spindle transfer is also known to be more difficult and less efficacious than pronuclear transfer (Reznichenko et al., 2016). Despite potential controversies, these techniques have been submitted for approval to begin testing in small cohorts of human patients. The goal of mitochondrial replacement techniques (MRT) is to provide patients with the chance to have unaffected biological children, through the reconstruction of functional oocytes and zygotes, to prevent the inheritance of mutated genes that result in primary mitochondrial diseases (Reznichenko et al., 2016).

Primary mitochondrial diseases can manifest at any point throughout an individual’s life, causing irreparable damage. With the lack of available curative treatments, currently, prevention is the only option for controlling inheritance in offspring, despite it being unreliable (Farnezi et al., 2020). The multiplication pattern of the mitochondria during embryonic development renders pre-implantation genetic diagnosis (PGD) unpredictable as the mtDNA burden for an embryo to develop with or without manifesting the disease is unknown (Farnezi et al., 2020). Additionally, patients with high penetrance of their particular mutation (i.e., highly homoplasmic, but abnormal mtDNA) cannot benefit from PGD, since all their offspring will most likely be affected (Farnezi et al., 2020). Due to the lack of success with PGD, MRTs have been investigated, originally initially proposed as a treatment for pregnancy loss, thought to be due to a post-fertilization energetic defect (Amato et al., 2014). Several techniques were developed in animal models to show different means of mtDNA transfer and to check viability in reconstructing functional oocytes and zygotes, while avoiding the inheritance of mutated genes (Craven et al., 2010). Considering the results from the animal model studies, MRTs were found to be a suitable alternative for patients with pathogenic mutations in mtDNA who wish to have unaffected offspring (Farnezi et al., 2020). Despite promising early results, there are still unanswered questions, including how to identify the amount of mtDNA to load during the procedure (Farnezi et al., 2020).

Despite these questions, the use of MRTs in humans, both pronuclear transfer and maternal spindle transfer, was approved in the United Kingdom on 29 October 2015, by the *Human Fertilisation and Embryology Act*, however, the first child was not born until 8 years later (Castro, 2016; Callaway, 2023). The UK has confirmed that fewer than five healthy babies have been born using MRTs since April 2023, however, the technique used in those cases was not disclosed (Castro, 2016). Alternatively, in the US, approval is managed by the US Food and Drug Administration, which, to date, has not granted permission for clinical use (Callaway, 2023).

Further, in light of (pending) approvals to use these techniques clinically, it is imperative to note that mismatches between mitochondrial and nuclear genomes may occur during this process (Dunham-Snary and Ballinger, 2015). While there are conflicting results, Deuse et al. demonstrated that the introduction of mitochondrial mismatched cells or tissues can be ineffective in producing viable offspring, as embryonic stem cells were unable to mature and transplanted tissue was immune to rejection (Deuse et al., 2015). In addition to challenges in the laboratory, these techniques face legal and ethical dilemmas regarding the involvement of the donor with the newly generated embryo (Bianco and Montagna, 2016). The resultant embryo will have distinct nuclear genetic material from the father (via the spermatozoon), the biological mother (specifically, her entire nuclear genetic complement) and complete mitochondrial genetic complement of the donor who provided an oocyte cytoplast, thus generating a “three-parent baby” (Farnezi et al., 2020). Given the rarity of application of these techniques, long-term effects in offspring generated via MRT are not known and additional investigation is necessary to further define and classify long-term effects and implications (Dunham-Snary and Ballinger, 2015; Farnezi et al., 2020).

### 2.3 CRISPR and gene editing in primary mitochondrial disease models

To avoid the various medical, ethical and potentially legal implications of MRTs, investigations into mitochondrial genome editing are ongoing (Pompei and Pompei, 2019). Recently, Reddy et al. employed the use of restriction endonucleases and mitochondrial transcription activator-like effector nucleases (mito-TALENs) to selectively eliminate mitochondrial genomes from a heteroplasmic mouse (Reddy et al., 2015). The NZB/BALB heteroplasmic mouse contains approximately equal amounts of both NZB and BALB mtDNA (Reddy et al., 2015). BALB mtDNA contains a unique restriction site that is not present in the NZB sequence, and by targeting this site using the ApaLI restriction enzyme, BALB mtDNA was successfully eliminated from oocytes as well as embryos (Reddy et al., 2015). This selective elimination was also performed to eliminate the NZB mtDNA that lacks restriction sites, instead using a mito-TALEN (Reddy et al., 2015). Targeted elimination using mito-TALENs has also been successful with human samples *in vitro*; mtDNAs harbouring one of the causal mutations of LHON were eliminated from a hybrid embryo (fusion of human cells carrying LHON mutation to a mouse MII oocyte) (Reddy et al., 2015). These findings suggest that, with additional investigation, techniques to eliminate pathogenic mtDNA mutations, while avoiding the legal and ethical implications of other methods, may be possible.

Other recent advancements, such as CRISPR/Cas9 technology, have revolutionized the ability to generate precise mitochondrial mutations *in vitro* and *in vivo* (Jo et al., 2015), enabling researchers to overcome limitations associated with earlier models, such as incomplete replication of human disease phenotypes. Gene-editing technologies, particularly CRISPR/Cas9 – gene editing technology that adds, removes, or changes DNA sections of the genome, have transformed the development of mitochondrial disease models by enabling precise manipulation of genetic sequences to replicate human disease-causing mtDNA mutations *in vivo*. CRISPR-mediated approaches have been employed to introduce or correct mtDNA mutations, thereby advancing our understanding of their functional consequences. For example, studies using CRISPR-based cytidine deaminase systems have successfully edited pathogenic mtDNA mutations in human cells, highlighting the therapeutic potential of these techniques (Bi et al., 2022; Schmiderer et al., 2022). However, ethical and safety considerations must be addressed before these technologies can transition to clinical applications *in vivo*.

Despite these advances, challenges remain such as production and labour costs; however, a significant barrier to success is understanding and developing a successful delivery mechanism. Due to variance in size of current delivery models and dependency of CRISPR on tRNAs, this has proven to be a major obstacle (Kolesnikova et al., 2004; Karicheva et al., 2011; Gammage et al., 2016; Gammage et al., 2018a; Bi et al., 2022; Schmiderer et al., 2022). However, recent innovations, such as mitochondrial-targeted base editors and RNA-guided nucleases, hold promise in overcoming these barriers, paving the way for more efficient and accurate mitochondrial gene editing (Yi et al., 2024). Moreover, while genetically modified models have enhanced our understanding of specific mutations, their relevance to multifactorial diseases and tissue-specific context remains limited. These challenges highlight the need for integrative approaches that combine the strengths of various model systems to achieve greater translational applicability.

### 2.4 Emerging *in vitro* systems: organoids and 3D cultures

The integration of organoid technologies (artificially grown 3D miniature version of an organ produced *in vitro*) has allowed for the study of mitochondrial dysfunction in a tissue-specific context, that mimics the key functional, structural, and biological complexity of the desired organ, thus offering novel insights into disease progression and therapeutic response (Pérez et al., 2021; Holubiec et al., 2023). This method allows for the manipulation and study of disease in isolated human-like organs, without the need to effect other organs and systems. The emergence of organoids and 3D culture systems represents a paradigm shift in preclinical modeling. These advanced *in vitro* systems recapitulate the structural and functional complexity of human tissues, providing a more physiologically relevant platform for studying mitochondrial diseases (Broutier et al., 2017; Brevini et al., 2020). Organoids derived from patient iPSCs have been used to model tissue-specific mitochondrial dysfunction, such as neuronal deficits in Leigh syndrome affecting key metabolic pathways (Romero-Morales et al., 2022 July 6). While these systems offer numerous advantages, including reduced reliance on animal models and enhanced translational potential, they are not without limitations. Challenges such as incomplete tissue maturation, lack of vascularization, and inter-organoid variability must be addressed to fully harness their potential (Qian et al., 2016; Wörsdörfer et al., 2020; Ye, 2023). Future research should focus on integrating organoid systems with microfluidic technologies and multi-omics approaches to enhance their fidelity and applicability.

### 2.5 Integration of multi-omics approaches in modeling disease

The integration of multi-omics approaches, encompassing genomics, transcriptomics, proteomics, metabolomics, and epigenomics, has revolutionized the study of mitochondrial-driven diseases. By providing a holistic view of cellular processes, multi-omics strategies have enhanced our ability to unravel the complex interplay between mitochondrial dysfunction and systemic pathophysiology (Piening et al., 2018; Rose et al., 2019; Zhou et al., 2019; Ranjbarvaziri et al., 2021; Liao et al., 2024). Studies have illustrated the power of these approaches in preclinical research. For instance, metabolomic profiling of mouse models with mtDNA mutations has identified biomarkers of oxidative stress and metabolic dysregulation, while proteomic analyses have elucidated mitochondrial protein interaction networks in neurodegenerative diseases (Graham et al., 2017). However, the integration and interpretation of multi-omics data remain challenging due to its high dimensionality and complexity (Subramanian et al., 2020; Odenkirk et al., 2021). Integrating expertise in involving bioinformatics, systems biology, and machine learning are essential to overcome challenges and fully realize the translational potential of multi-omics approaches in mitochondrial disease research.

## 3 Implications for disease understanding, therapeutics, and future directions

### 3.1 Translational potential of preclinical models

Preclinical models have profoundly advanced our understanding of the molecular and cellular mechanisms underlying mitochondrial diseases. As previously discussed, these models have been pivotal in uncovering the role of OXPHOS dysfunction, ROS overproduction, and impaired mitochondrial dynamics in driving disease pathology (Ng and Turnbull, 2016; Bhatti et al., 2017; Chen et al., 2023; Choi et al., 2024). For instance, rodent models with mutations in nuclear-encoded *POLG*, which encodes the mitochondrial DNA polymerase, have elucidated the link between defective mtDNA replication and cardiac dysfunction (Lewis et al., 2007). Similarly, studies using induced pluripotent stem cell-derived neurons have highlighted the cell-type-specific vulnerabilities to mitochondrial dysfunction observed in disorders such as Leigh syndrome and MELAS (Chichagova et al., 2017; Galera-Monge et al., 2020; Inak et al., 2021; Bhattacharya et al., 2022), and diseases such as Alzheimer’s and Parkinson’s (Cooper et al., 2012; Flannagan et al., 2023). The identification of novel pathways, such as the role of mitophagy and mitochondrial biogenesis in disease progression, has opened new avenues for therapeutic intervention. For example, targeting the PINK1-Parkin pathway to regulate mitophagy has shown promise in preclinical models of MELAS (Hämäläinen et al., 2013; Pickrell and Youle, 2015). These insights not only contribute to the development of targeted therapies but also enhance our broader understanding of mitochondrial biology and its systemic implications.

Preclinical models of mitochondrial-driven diseases have demonstrated immense potential in bridging basic research and clinical application. Their utility extends from elucidating disease mechanisms to identifying and validating therapeutic strategies. For example, mouse models of mitochondrial myopathy, such as those harboring mutations in the mitochondrial tRNA synthetase genes, have been instrumental in understanding disease progression and testing therapeutic interventions, including gene therapy^,^ as previously discussed. Similarly, patient-derived induced pluripotent stem cells have enabled researchers to model mitochondrial dysfunction in a human cellular context, revealing novel drug targets and potential biomarkers (Prigione, 2015; Luo et al., 2022).

Despite advances, the translational success of preclinical models remains inconsistent due to species-specific differences in mitochondrial biology and an inability to fully recapitulate the clinical heterogeneity of mitochondrial diseases. Addressing these limitations requires a more integrative approach that combines advanced *in vitro* systems with *in vivo* studies to enhance predictive accuracy. Collaboration among academic institutions, industry stakeholders, and regulatory agencies will be essential in bridging this gap and translating preclinical findings into patient care.

The complexity of mitochondrial diseases necessitates a collaborative, cross-disciplinary approach to research. Partnerships between clinicians, molecular biologists, bioengineers, and data scientists can accelerate the development and validation of innovative models. Notable examples include collaborations between institutions and pharmaceutical companies to streamline drug discovery pipelines for mitochondrial disorders and personalized medicine strategies Interdisciplinary groups, such as the *Mitochondrial Medicine Society* and the *International Mito Patients organization*, provide valuable platforms for knowledge exchange and collective problem-solving (MitoCanada, 2022). Future initiatives should aim to expand these networks, incorporating diverse expertise and perspectives from global stakeholders. Cross-sector collaborations, particularly between academia and industry, hold immense potential for fostering innovation. For example, the co-development of organ-on-chip systems by academic researchers and biotechnology firms has already shown promise in advancing translational applications (Huh et al., 2010; Singh et al., 2022). Funding bodies and policymakers should prioritize investments in collaborative projects that bridge basic and applied research, ensuring a sustainable pipeline for mitochondrial disease innovations.

The field of mitochondrial disease modeling is experiencing a transformative phase driven by advancements in bioengineering, gene-editing technologies, and computational modeling. Emerging trends include the development of more sophisticated *in vitro* systems, such as patient-derived organoids and 3D cultures that closely mimic the structural and functional characteristics of human tissues. These models offer unprecedented insights into tissue-specific mitochondrial pathologies and therapeutic responses. Recent breakthroughs in single-cell technologies and spatial transcriptomics are providing high-resolution views of mitochondrial dysfunction at the cellular and subcellular levels (Lareau et al., 2021; Schäfer et al., 2023; Ya et al., 2023). Similarly, organ-on-chip platforms integrated with microfluidics are enabling dynamic studies of mitochondrial biology within a physiologically relevant microenvironment (Abudupataer et al., 2021; Fuchs et al., 2022; Li et al., 2022). For example, Abudupataer et al. used a microfluidics-based aorta smooth muscle-on-a-chip model to show that mitochondrial fusion activators or fission inhibitor can partially rescue the disorders of mitochondrial dynamics in human aorta smooth muscle cells derived from patients with the potential to develop thoracic aortic aneurysms due to a bicuspid aortic valve (Abudupataer et al., 2021). These systems hold promise for investigating disease heterogeneity and drug responses in a controlled yet complex setting. Looking forward, the integration of artificial intelligence and machine learning in mitochondrial research is expected to enhance the predictive power of preclinical models. These computational tools can streamline data analysis, uncover novel mitochondrial pathways, and refine drug screening pipelines (Abbas et al., 2024). Collaborative efforts should focus on validating these emerging technologies and incorporating them into existing research frameworks to maximize their impact on the field.

### 3.2 Role of models in drug discovery and development

The development of effective therapeutics for mitochondrial diseases relies heavily on the insights gained from preclinical models. These models serve as platforms for the identification and validation of drug candidates, enabling high-throughput screening and functional testing. For instance, zebrafish models with mitochondrial dysfunction have been utilized to test current FDA-approved drugs, leading to the discovery of compounds that can ameliorate oxidative stress and restore mitochondrial function in Leigh Syndrome (Haroon et al., 2023). Kayser et al. showed that the vitamin E analog vatiquinone (EPI-743) was able to prevent ferroptotic cell death *in vitro*, and may prevent seizures, in mouse models of Leigh syndrome (Kayser et al., 2025). In a metabolic syndrome model, the GLP-1 receptor agonist exendin-4 was given to hyperglycemia mice, resulting in sustained plasma glucose reduction, weight loss, and enhances insulin sensitivity over multiple weeks of treatment (Young et al., 1999).

One notable example of drug development pipelines influenced by preclinical findings include the advancement of elamipretide, a peptide designed to stabilize mitochondrial membranes, which showed efficacy in preclinical models of Barth syndrome and Primary Mitochondrial Myopathy and subsequently progressed to clinical trials (Sabbah, 2022; Karaa et al., 2023). However, limitations in predicting drug efficacy and safety remain a significant hurdle. Many compounds that demonstrate promise in preclinical models struggle to replicate these results in clinical settings due to differences in metabolic pathways, immune responses, and disease progression between model organisms and humans.

Addressing these limitations requires the incorporation of more physiologically relevant models, such as humanized animal models and organ-on-chip technologies, into the drug development pipeline. Additionally, leveraging multi-omics data to refine drug target selection and using computational modeling to predict drug responses could enhance the success rate of translating preclinical findings into effective therapies.

### 3.3 Challenges in predicting clinical outcomes

Discrepancies between preclinical and clinical outcomes remain a persistent challenge in mitochondrial research, driven by differences between model systems and human pathophysiology. Rodent models, for example, often fail to replicate human metabolic demands, disease progression, and therapeutic responses (Rossmann et al., 2021). Key limitations include oversimplified disease models, inadequate genetic and phenotypic representation, and challenges in assessing long-term outcomes. Advancing translational accuracy requires sophisticated models like 3D organoids and patient-derived xenografts, integrated with advanced imaging and bioinformatics. A multidisciplinary approach - combining genomics, proteomics, and metabolomics with experimental validation–and stronger collaboration among researchers, clinicians, and industry will be critical in transforming preclinical findings into clinical outcomes, ultimately driving the development effective treatments for mitochondrial-driven diseases.

### 3.4 Unmet needs and gaps in research

Current research efforts in mitochondrial disease modeling often focus on well-characterized disorders, such as MELAS, LHON, and neurodegenerative diseases, leaving rarer and less understood conditions underexplored. Addressing these gaps requires targeted funding and research priorities that incentivize the study of neglected mitochondrial pathologies. Another pressing need is the development of models that capture the multifactorial nature of secondary pathologies, which arise from systemic diseases such as diabetes, cancer, and neurodegeneration. These models must account for the interplay between mitochondrial defects and broader physiological systems, highlighting the importance of systems-level approaches. Efforts should also focus on improving the scalability and accessibility of advanced model systems. Cost-effective alternatives to organoids and 3D cultures, as well as standardized protocols for their generation and use, are crucial for ensuring reproducibility of results and equitable research opportunities across institutions with varying resources.

Building on these priorities, mitochondria-related DNA-driven mechanisms offer a rich avenue for future investigation. At the heart of this effort lies precise interrogation of how specific mtDNA variants, ranging from point mutations to large scale deletions, alter organelle function, intergenomic communication, and cellular metabolism. Advances in single-molecule sequencing and long-read technologies now allow high-resolution mapping of heteroplasmic landscapes across tissues, while emerging CRISPR-based base editors and targeted nucleases promise locus-specific correction of pathogenic variants without invoking double-strand breaks. Strategies such as precise manipulation of heteroplasmy levels, using mitochondrially targeted nucleases (mitoTALENs, mitoZFBs) or base editors, can elucidate the threshold at which mutant mtDNA drives cell dysfunction. Emerging epigenetic editing approaches promise to modulate mtDNA copy number or methylation *in situ*, thereby tuning organelle performance. High-throughput single-cell sequencing and imaging will map heteroplasmy dynamics across tissues, informing lineage-specific susceptibilities and therapeutic windows. Complementing these wet-lab advances, AI-driven *in silico* models of mtDNA replication, repair, and transcription are poised to predict the functional impact of novel variants and optimize guide RNAs for mitochondrial CRISPR-free editing platforms. Together, these DNA-centric trajectories will deepen mechanistic insights and pave the way for precision mitochondrial medicine.

## 4 Conclusion

This review has aimed to highlight the critical role of preclinical models in advancing our understanding of mitochondrial-driven diseases. Various mtDNA models have been developed over the past 50 years, each largely contributing to revolutionary discoveries, while illuminating both the strengths and the limitations of its predecessors. Refinements have been made to each model as research continued to strive towards identifying the optimal way to investigate the isolated effects of mtDNA signature with the ultimate goals of application of the techniques to a human population. Building on pioneering cybrid cell studies that underscored the need for whole-organism models of mitochondrial disease, researchers next developed xenomitochondrial and conplastic animals. These were elegant platforms that successfully bridged the gap between cellular phenotypes and *in vivo* pathophysiology, allowing for the exploration of disease progression at the tissue and organismal levels. To achieve uniform mitochondrial genomes, the field then adapted and the MNX mouse model was introduced, which uses pronuclear transfer to establish complete homoplasmy and enable precise dissection of mitochondrial-nuclear interactions in health and disease. Building on this success, maternal spindle transfer was used to generate homoplastic non-human primate models, demonstrating the broad applicability of MNX technology across species. These breakthroughs have now paved the way for carefully regulated “three-parent” clinical approaches in the UL and US, offering new hope for families affected by mitochondrial diseases. Looking ahead, advanced mitochondrial genome-editing strategies promise to refine these therapies further, potentially delivering safe, one-step cures without the need for donor mitochondria.

Recent innovations, such as patient-derived organoids, gene-editing technologies, and organ-on-chip platforms, have provided unprecedented insights into the molecular underpinnings of mitochondrial dysfunction. These advancements have expanded our understanding of disease mechanisms, identified novel therapeutic targets, and contributed to the refinement of drug discovery pipelines. Despite these achievements, the translational gap between preclinical findings and clinical outcomes remains a significant challenge. This underscores the need for robust models that capture the complexity of human mitochondrial diseases and the importance of interdisciplinary collaboration in addressing these limitations. For researchers and clinicians, the advancements discussed here emphasize the necessity of integrating cutting-edge tools and collaborative frameworks to optimize both experimental outcomes and translational impact.

Moving forward, several strategic priorities must guide mitochondrial disease modeling. Enhancing model fidelity through the integration of patient-derived systems and humanized animal models is paramount. Addressing underexplored areas, such as secondary pathologies and rare mitochondrial disorders, will fill critical knowledge gaps and broaden the applicability of preclinical findings. Moreover, adopting cross-disciplinary approaches can help refine models, making them more predictive of clinical outcomes. Equally important is the need for scalable and accessible model systems that can inform mitochondrial research globally. The development of standardized protocols for advanced models will also improve reproducibility and facilitate broader adoption within the scientific community. The implications of this research extend far beyond the laboratory. The insights and technologies generated through advanced modeling systems have the potential to revolutionize clinical care for patients with mitochondrial diseases. By bridging the translational gap, these models can accelerate the development of targeted therapies and personalized treatment strategies, ultimately improving patient outcomes.

In the long term, the integration of these advancements into clinical practice could redefine the management of mitochondrial diseases. From early diagnostics to precision therapeutics, the future of mitochondrial medicine is set to be transformative. However, realizing this vision requires a collective effort from researchers, clinicians, industry leaders, and policymakers to foster innovation and ensure equitable access to emerging therapies. The continued evolution of mitochondrial disease modeling represents a critical frontier in translational medicine. By leveraging emerging technologies, addressing unmet research needs, and fostering collaborative frameworks, the field can achieve its ultimate goal: improving the lives of patients affected by these complex and often devastating disorders.

## Abbreviations

CMD, Cardiometabolic Disease; ETC, Electron Transport Chain; ES, Embryonic Stem; LHON, Leber’s Hereditary Optic Neuropathy; MELAS, Mitochondrial Encephalopathy, Lactic Acidosis, and Stroke-Like Episodes; MNX, Mitochondrial Nuclear eXchange; MRT, Mitochondrial Replacement Therapy; NGS, Next-Generation Sequencing; OXPHOS, Oxidative Phosphorylation; PGD, Preimplantation Genetic Diagnosis; ROS, Reactive Oxygen Species.
